# Optical design of the Short Pulse Soft X-ray Spectroscopy beamline at the Advanced Photon Source

**DOI:** 10.1107/S0909049513013149

**Published:** 2013-05-30

**Authors:** R. Reininger, D. J. Keavney, M. Borland, L. Young

**Affiliations:** a Advanced Photon Source, Argonne National Laboratory, Argonne, IL 60439, USA

**Keywords:** picosecond X-ray pulses, soft X-ray beamline, time-resolved spectroscopy, VLS grating

## Abstract

The Short Pulse X-ray facility planned for the Advanced Photon Source (APS) upgrade will provide two sectors with photon beams having picosecond pulse duration. The Short Pulse Soft X-ray Spectroscopy (SPSXS) beamline will cover the 150–2000 eV energy range using an APS bending magnet. SPSXS is designed to take full advantage of this new timing capability in addition to providing circular polarized radiation. Since the correlation between time and electron momentum is in the vertical plane, the monochromator disperses in the horizontal plane. The beamline is designed to maximize flux and preserve the time resolution by minimizing the number of optical components. The optical design allows the pulse duration to be varied from 1.5 to 100 ps full width at half-maximum (FWHM) without affecting the energy resolution, and the resolution to be changed with minimal effect on the pulse duration. More than 10^9^ photons s^−1^ will reach the sample with a resolving power of 2000 and a pulse duration of ∼2 ps for photon energies between 150 and 1750 eV. The spot size expected at the sample position will vary with pulse duration and exit slit opening. At 900 eV and at a resolving power of 2000 the spot will be ∼10 µm × 10 µm with a pulse duration of 2.3 ps FWHM.

## Abstract 2

The optical design of the SPSXS beamline at the APS is described. The beamline will allow time-resolved experiments in the picosecond range with moderate resolution and relatively high linear and circular polarized flux.

## 1. Introduction   

The Short Pulse Soft X-ray Spectroscopy (SPSXS) beamline is one of the beamlines planned for the upgrade of the Advanced Photon Source (APS) at Argonne National Laboratory which will occur during 2013–2020. This beamline will make use of the short-pulse X-ray (SPX) source: the implementation of the deflecting-cavity scheme proposed by Zholents *et al.* (1999 ▶) for the production of tunable few-picosecond soft and hard X-ray pulses at the 6.5 MHz repetition rate of the APS. The other two beamlines that take advantage of the SPX source produce hard X-rays, the Short Pulse X-ray Scattering and Spectroscopy (SPXSS) and the Short Pulse X-ray Imaging and Microscopy (SPXIM) (Reininger *et al.*, 2013 ▶) beamlines. These three beamlines are located between the two RF deflection cavities (Zholents *et al.*, 1999 ▶; Borland, 2005 ▶) that provide chirped electron pulses. The propagation of the APS storage ring’s electron beam through the cavities imposes a correlation between the longitudinal position of an electron in the bunch and its vertical momentum. The X-ray radiation emitted during the electron propagation through an undulator (for SPXSS and SPXIM) or a bending magnet (6BM for SPSXS) preserves the correlation and a short pulse can be selected out with slits. The SPX facility will be equipped with high-power and high-repetition-rate (up to 6.5 MHz) femtosecond lasers, similar to those already existing at the Advanced Photon Source (March *et al.*, 2011 ▶).

Two soft X-ray beamlines using the slicing technique proposed by Zholents & Zolotorev (1996 ▶) for obtaining pulses with sub-100 fs time resolution are operational, one at the BESSY II storage ring (Khan *et al.*, 2006 ▶) and the other at the Advanced Light Source (Heimann *et al.*, 2007 ▶). Both beamlines use radiation from an undulator. Owing to the low yield of the slicing method, they provide up to 10^6^ photons s^−1^ (0.1% bandwidth)^−1^ to the sample in a ∼100 fs pulse. This relatively high flux from a slicing source at the beamline at BESSY is due to a recently implemented very efficient spectrometer based solely on a reflection zone plate (Erko *et al.*, 2010 ▶) and is optimized for polarization-dependent spectroscopy.

The operation of BESSY II in a low-momentum compaction lattice (low α mode) demonstrated the capability of reducing the bunches to picosecond duration (Feikes *et al.*, 2004 ▶). Similar results were obtained later at SPEAR3 (Corbett *et al.*, 2009 ▶). The disadvantage of the low α mode of operation is the significant lower current in each bunch and consequently the lower overall stored current, which is detrimental for users interested in high flux and not picosecond pulses. Therefore, the low α mode of operation, bunches with less than 0.1 mA and pulse durations between 2 and 5 ps (FWHM), is run at BESSY II only four times a year for three days.

Despite using a bending magnet as a source, the soft X-ray flux delivered to the sample by the SPSXS beamline will be significantly higher than at facilities operating a slicing mode or a low α mode. However, the time resolution available at SPSXS will be in the few picosecond range as compared with the ∼100 fs range obtained by slicing.

Time-resolved magnetic spectroscopy on the picosecond time scale is ideally matched to dynamics in many materials where a close coupling between electronic, magnetic and structural degrees of freedom leads to the emergence of new functional properties such as magnetoelectric and magnetoelastic behavior, spin-polarized transport and metal–insulator transitions. The ability to directly couple laser fields to specific structural and electronic excitations and follow their magnetic response with element, time and spatial resolution offers a powerful way to understand the origins of material properties at a fundamental level. Materials that may display such rich phenomenology, *e.g.* complex oxides, highly correlated electron systems, coupled multilayer systems and ordered metallic alloys, will be a strong component of the early science at SPSXS. Consequently, the SPSXS beamline instrumentation will be geared toward magnetic spectroscopy in transmission, fluorescence and reflectivity in the energy range 150–2000 eV, where many of these materials have important absorption edges. The experimental program requires linear and circular polarization, moderate resolving power (*E*/Δ*E* = 1000 to 5000), and a pulse duration between 1 and 100 ps full width at half-maximum (FWHM). An interchangeable two-dimensional area-detector/exit-slit assembly will provide functionality for transmission spectroscopy in a dispersive geometry or monochromatic spectroscopy in an experimental chamber equipped with a high-field magnet downstream of the exit slit.

In the sections below we describe the electron and photon source, the optical design and the expected performance of the SPSXS beamline as well as ray tracings verifying the analytical calculations.

## 2. Source   

Soft X-ray experiments at the SPX facility will be conducted utilizing a bending-magnet source since soft X-ray undulators with the chirped electron beam present significant technical problems. Most notably, the energy dependence of the pulse duration limits the achievable performance to 8–10 ps (FWHM) with 2 MV acceleration in the deflecting cavities (Emery *et al.*, 2011 ▶).

**Figure 1:**
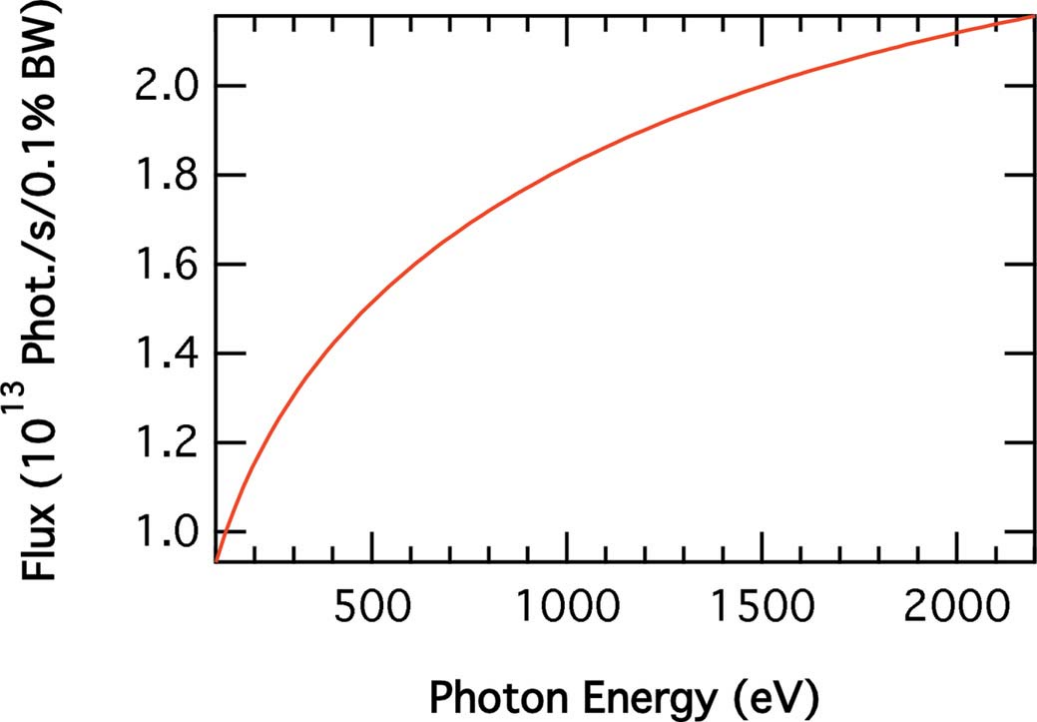
Flux from a standard APS bending magnet. The current in the storage ring is 150 mA and the horizontal angle is 1 mrad.

**Figure 2:**
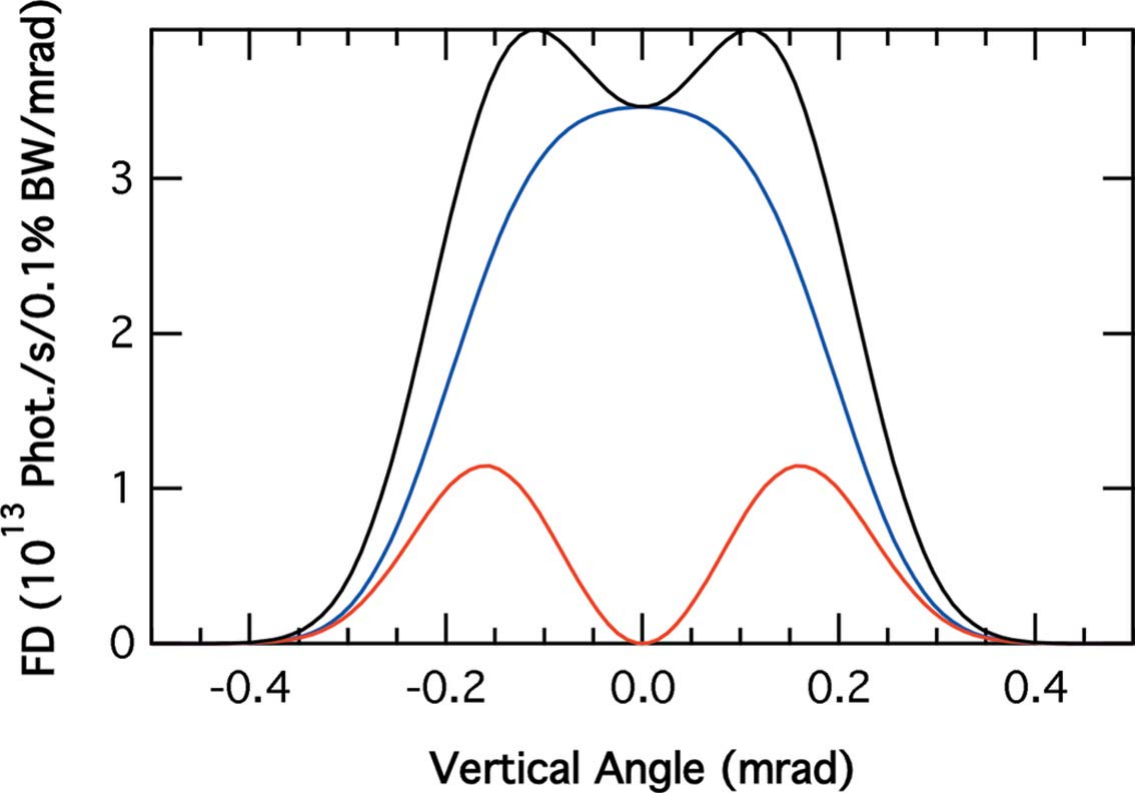
Vertical distribution of the flux for horizontal (blue), vertical (red) and total polarization (black) emitted by a standard APS bending magnet at 850 eV. The current in the storage ring is 150 mA.

Fig. 1 ▶ shows that the total flux emitted over 1 mrad by a standard APS bending magnet (0.6 T) in the 100–2200 eV range with 150 mA stored in the ring is more than 10^13^ photons s^−1^ (0.1% bandwidth)^−1^. The high critical energy of the APS bending magnet, 19.5 keV, means that the vertically polarized radiation emitted off-axis by a bending magnet at soft X-ray energies is relatively large (see Fig. 2 ▶). Furthermore, at these energies one can obtain high averaged circular polarized (CP) radiation with high flux percentage (FP) and consequently high figure of merit [CP × (FP)^1/2^] (Chen, 1992 ▶). Collecting the radiation emitted by the bending magnet between 0.05 and 0.5 mrad vertically gives 86% circular polarized radiation, 40% of the flux and a figure of merit equal to 0.54 at 850 eV. Reducing the acceptance to between 0.1 and 0.5 mrad at the same photon energy increases the percentage of circular polarization to 93%, reducing the accepted flux and figure of merit to 29% and 0.5, respectively.

The statistical representation of the radiation at a given photon energy was obtained in two steps. In the first step, the accelerator simulation code *ELEGANT* (Borland, 2000 ▶) is used to determine the equilibrium properties of 10^6^ electrons at the bending-magnet position, sampling the horizontal and vertical phase space as well as time (position along the electron bunch). These calculations were performed assuming 24-bunch mode with a 41 ps RMS electron bunch length. In the second step, the electron distribution was convoluted with a statistical sampling of bending-magnet radiation at the required photon energy on a 1 mrad × 1 mrad aperture obtained using the *SHADOW* code (Welnak *et al.*, 1994 ▶; Sanchez del Rio *et al.*, 2011 ▶) assuming an electron beam with negligible horizontal and vertical emittance. The composite source is then used in the ray tracings described in §3.

**Figure 3:**
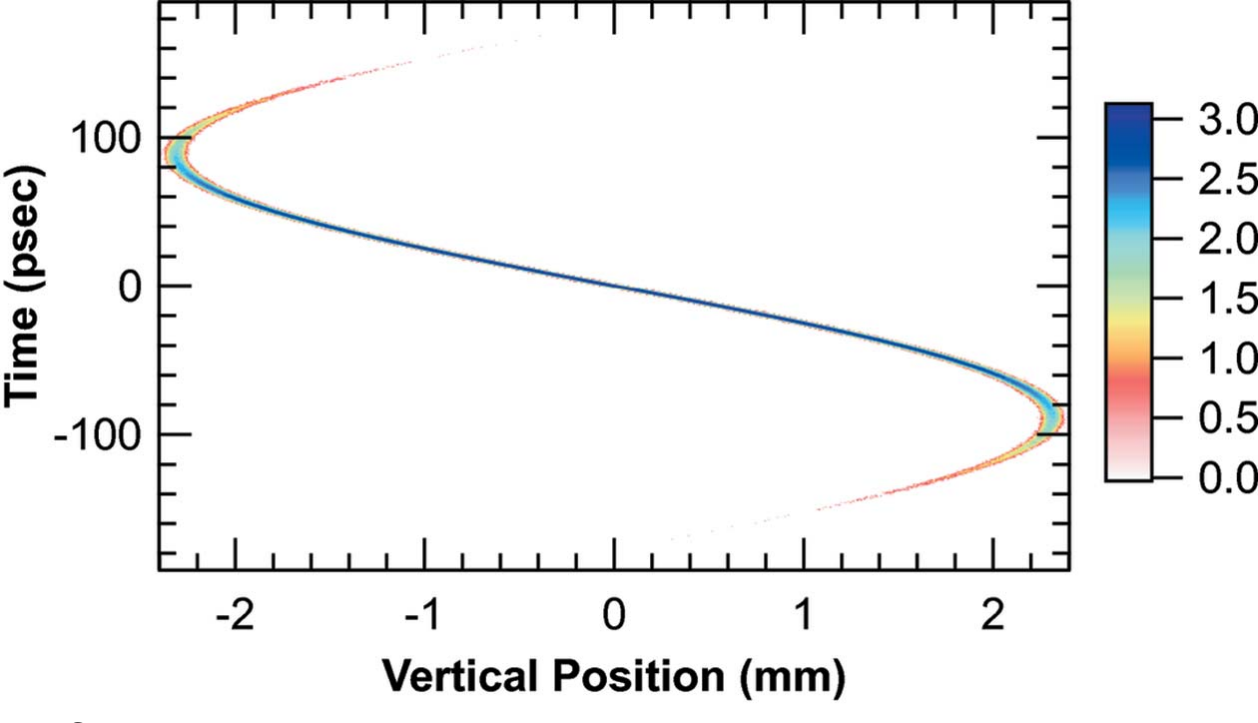
Log (base 10) of the number of electrons as a function of time and vertical position (see text).

Fig. 3 ▶ shows the correlation between time and vertical position of the electron beam at the bending magnet. The slope obtained from the linear region is 25 ps mm^−1^ and the time resolution (in the limit of zero height) is 1.3 ps FWHM. A logarithmic representation of the 10^6^ particles in the figure is chosen to illustrate the correlation and show the significant variation in the number of particles displayed. We note in passing that the ‘back-chirp’ seen in the correlation is caused by the fact that the accelerating half-period in the cavity (178 ps) is shorter than the electron bunch.

**Figure 4:**
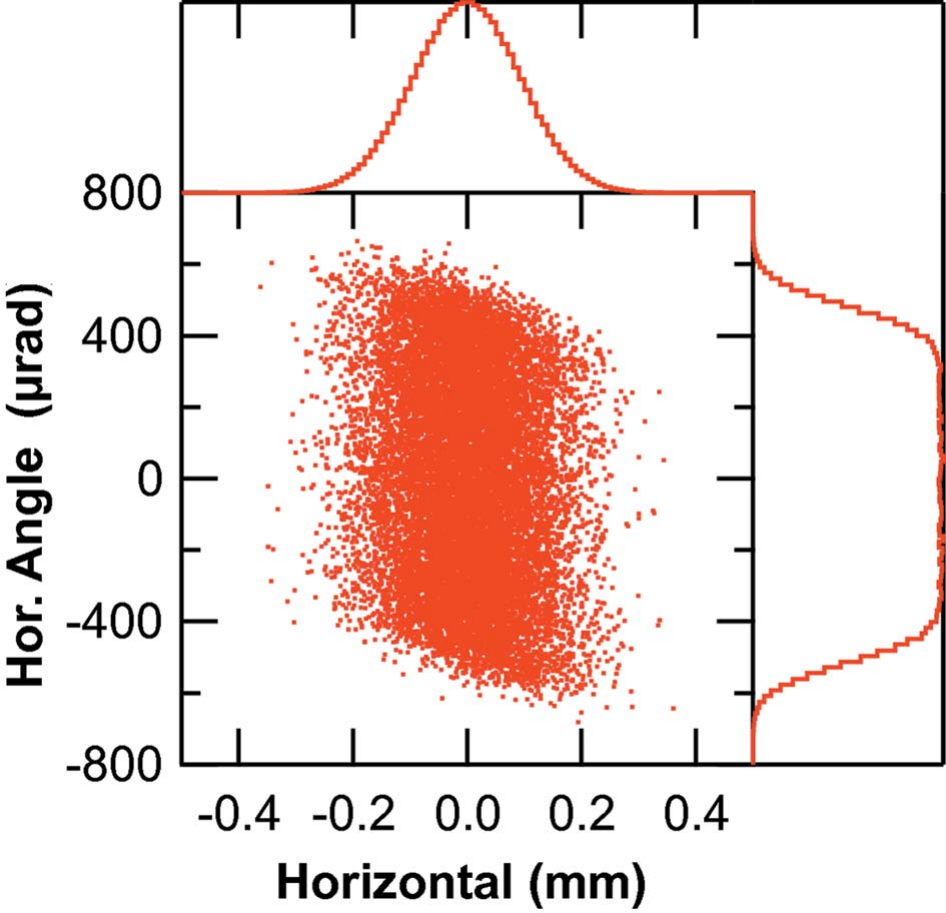
Horizontal phase space of the bending-magnet radiation at 900 eV due to the chirped electron pulse. The number of rays in the figure has been reduced to prevent complete saturation. The right-hand histogram shows the distribution in angle and the top histogram the distribution in position.

**Figure 5:**
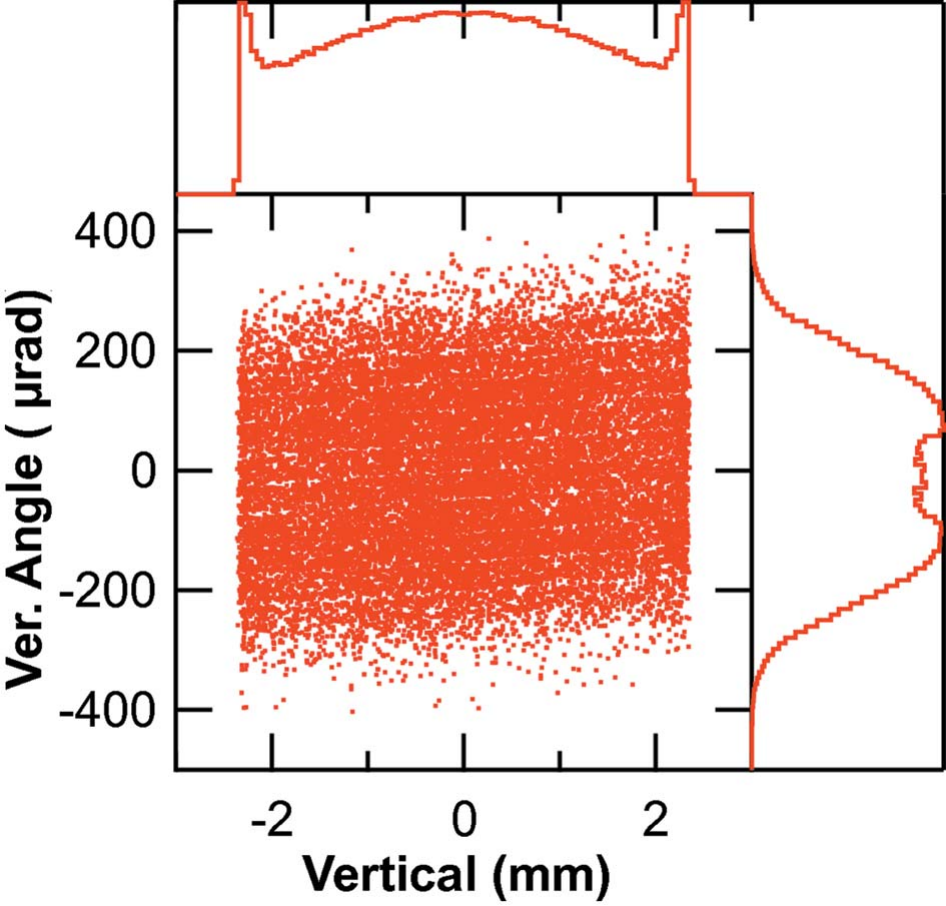
Vertical phase space of the bending-magnet radiation at 900 eV due to the chirped electron pulse. The number of rays in the figure has been reduced to prevent complete saturation. Histograms are as in Fig. 4 ▶.

The horizontal and vertical phase spaces of the convoluted chirped electron bunch with the radiation of the bending magnet at 900 eV is shown in Figs. 4 ▶ and 5 ▶, respectively. The photon beam horizontal size (Fig. 4 ▶) is dominated by the electron beam size and has a FWHM of 220 µm whereas the horizontal angle of the radiation is actually set by the acceptance of the first ellipsoidal mirror. The vertical size of the photon beam (Fig. 5 ▶) is fully determined by the chirped electron pulse and its histogram shows the two spikes resulting from the ‘back-chirp’. The histogram of the distribution of the vertical angles is actually that of the 900 eV radiation and it reproduces the total distribution displayed in Fig. 2 ▶.

## 3. Optical layout   

**Figure 6:**
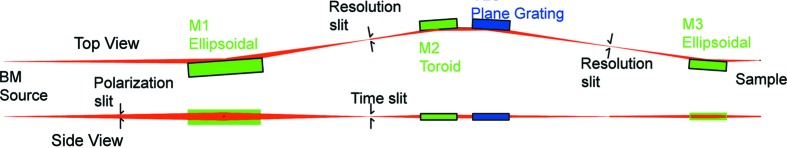
Optical layout of the SPSXS beamline.

Fig. 6 ▶ shows the optical layout of the SPSXS beamline. Table 1 ▶ summarizes the position of the optical elements.

The main consideration in the optical design of the SPSXS beamline is to maximize the flux at the experiment station with medium resolving power (up to *E*/Δ*E* = 5000) without impairing the time resolution. As described below, this is achieved with only four optical elements, all deflecting the beam in the horizontal plane. The SPSXS beamline will exploit the vertical timing dispersion inherent in the RF-chirped bending-magnet source and use horizontal dispersion for photon energy selection. This requires a focusing system in the first optics enclosure that will illuminate a horizontal slit for energy resolution selection and a vertical slit for timing resolution selection.

A movable vertical aperture upstream of the first optical element will select radiation from above or below the plane providing a relatively high percentage of circular polarization as described in §2.

A Kirkpatrick–Baez (KB) mirror pair with elliptical cylinders is the optimal optical configuration in terms of image quality for focusing the bending-magnet source onto the timing and resolution slits. However, the requirement for the minimum number of mirrors, to reduce reflectivity losses, and a beam parallel to the floor at the experimental stations implies that an additional mirror with a vertical deflection will be necessary if a KB system is chosen.

Floor space constraints dictate a demagnification on the first mirror of approximately 2.5. This demagnification, when combined with the relatively long mirror required (800 mm), rules out the use of a toroidal mirror due to the resulting large coma aberration.

The use of a horizontally deflecting ellipsoidal mirror with meridional RMS slope errors of up to ∼1 µrad is sufficient to avoid losses at the horizontal (energy) slit since its size (2 × 2.35 × 10^−6^ × 24.8 m = 117 µm) at the source plane is relatively small (combined in quadrature) compared with the horizontal beam size (FWHM of 220 µm). A RMS sagittal slope error of 10 µrad on this mirror translates to a pulse duration of approximately 2 × 2.35 × 10^−5^ × tan(1.5°) × 24.8 m × 25 ps mm^−1^ = 0.76 ps, which will worsen the time resolution from 1.3 ps to 1.5 ps (FWHM). Based on these considerations and the feasibility of manufacturing such a mirror we opted for an ellipsoidal mirror as the element imaging the source onto the (energy) resolution and timing slits.

Two orthogonal slits will be placed at a distance of 10 m downstream of the ellipsoidal mirror. The timing slit (vertical) will select the pulse duration and will be adjustable to accept 1–100% of the incident radiation, providing pulse durations of 1.5–100 ps. The horizontal slit will serve as the monochromator entrance slit. The slit assembly will be included in an experiment station for transmission spectroscopy of optically thin samples. The sample space will be immediately downstream of the slit assembly to minimize the beam size.

For the monochromator we chose a variable-line-spacing (VLS) plane-grating (PG) monochromator (Hettrick, 1988 ▶; Amemiya *et al.*, 1996 ▶; Reininger *et al*., 2004 ▶) working in converging light. The gratings’ line density variation, *k*, as a function of the position, *w*, along the grating is given by *k*(*w*) = *k*_0_ + *a*_1_ *w* + *a*_2_ *w* ^2^ + *a*_3_ *w* ^3^, where *k*_0_ is the line density at the grating center. The coefficients *a*_1_, *a*_2_ and *a*_3_ are chosen to zero the defocus, the coma and the spherical aberration of the mirror–grating combination at one photon energy (Amemiya *et al.*, 1996 ▶). The parameters of the line density variation are listed in Table 2 ▶ (*w* is positive towards the exit slit). For the focusing element upstream of the grating, which provides converging light, we chose a horizontally deflecting toroid that focuses the radiation both horizontally and vertically at the exit slit plane, *i.e.* when tuned to zero order the monochromator focuses the zero order. The VLS PG monochromator focuses the virtual horizontal source (at the exit plane) onto the horizontal exit slit which will be interchangeable with the two-dimensional area detector located at the exit slit plane for collection of energy- (horizontal plane) and time-dependent (vertical plane) spectra.

The final optical component of the beamline is a second ellipsoidal mirror which demagnifies the horizontal exit slit and the vertical size of the beam at the exit slit plane by a factor of four onto the sample located 1 m downstream.

## 4. Expected performance and ray tracings   

In the simulations and ray tracings presented below we have assumed achievable RMS meridional (sagittal) slope errors of 1 µrad (10 µrad) on the ellipsoidal mirrors, 0.5 µrad (10 µrad) in the toroid and 0.2 µrad (0.5 µrad) on the plane grating.

**Figure 7:**
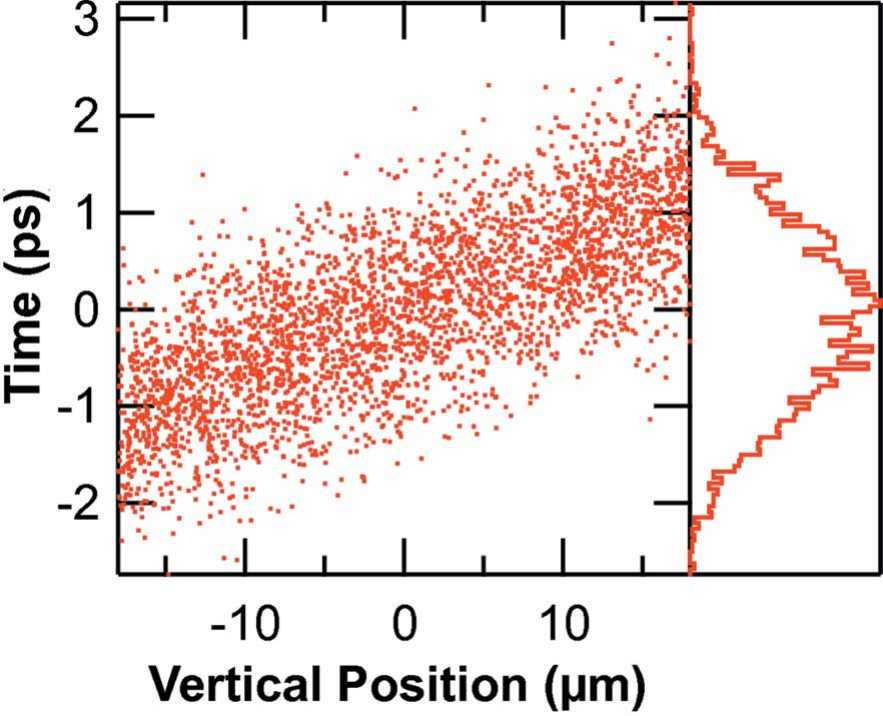
Correlation between time and vertical position at the entrance slit. The right-hand histogram shows the distribution in time.

Fig. 7 ▶ shows a *SHADOW* simulation at the timing slit when it is set to 36 µm. The correlation between time and vertical position at the vertical slit plane is 62 ps mm^−1^, 2.5 times more than that of the source, as expected from the mirror demagnification. Based on these values one would expect a square pulse length of 2 ps. Actually, the time resolution of the source and the slope errors on the mirror cause a broadening to a pulse having a FWHM of 2.3 ps as shown in the histogram in the figure. The time resolution will also be affected by the time broadening caused by the number of illuminated lines of the grating (*N*). This contribution, which is equal to *N*λ/*c*, where λ is the wavelength and *c* the speed of light, needs to be summed in quadrature with the contributions due to the time slit and slope errors to obtain the total time resolution.

**Figure 8:**
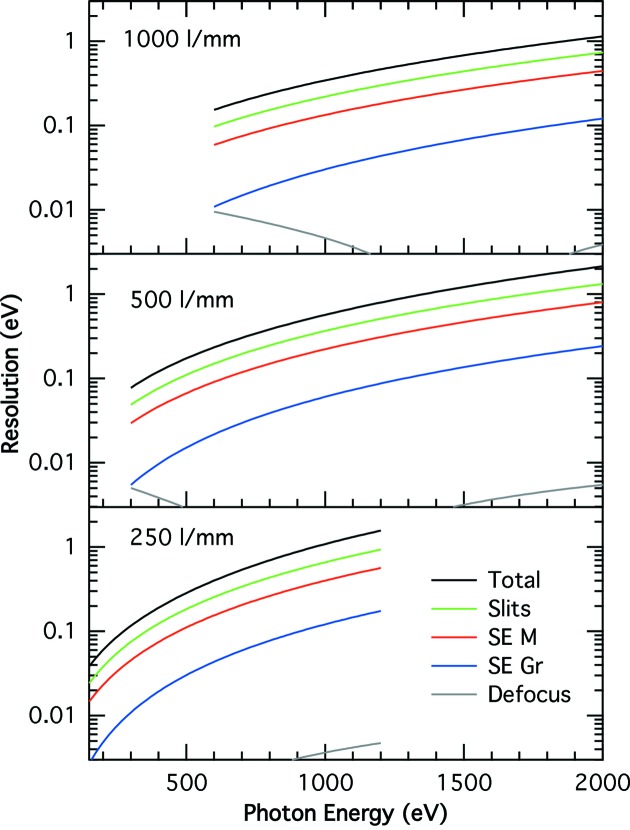
Resolution due to defocus (gray line), slope errors on grating (blue line) and toroidal (red line), entrance and exit slits (green line), and their total (black) for gratings with line densities at their centers of 250 lines mm^−1^ (bottom figure), 500 lines mm^−1^ (middle figure) and 1000 lines mm^−1^ (top figure).

Three VLS gratings were chosen to cover the full energy range of the beamline (Table 2 ▶). The low-energy grating (LEG) has a line density of 250 lines mm^−1^ at its center and was optimized to zero the defocus, coma and spherical aberration at 400 eV. The corresponding values for the medium-energy grating (MEG) are 500 lines mm^−1^ and 900 eV, and for the high-energy grating (HEG) 1000 lines mm^−1^ and 1500 eV. The contributions to the energy resolution of the defocus, slope errors on the toroid and the grating, the contribution of the entrance and exit slit as well as the square root of the sum of their squares (total in the figure) are displayed in Fig. 8 ▶. The contributions of the coma and spherical aberrations to the energy resolution are negligible as compared with that of the defocus for the three gratings which, as seen in the figure, is less than 10 meV for the three gratings. The entrance slit in all cases shown in the figure is set to 20 µm and the exit slit is set to obtain the same resolution as that of the entrance slit. Under this condition the exit slit width varies from 220 µm at the lowest energy of each grating down to a minimum of 23 µm when using the LEG at 1.2 keV. We note in passing that smaller slope errors on the toroidal mirror will decrease its energy resolution linearly.

**Figure 9:**
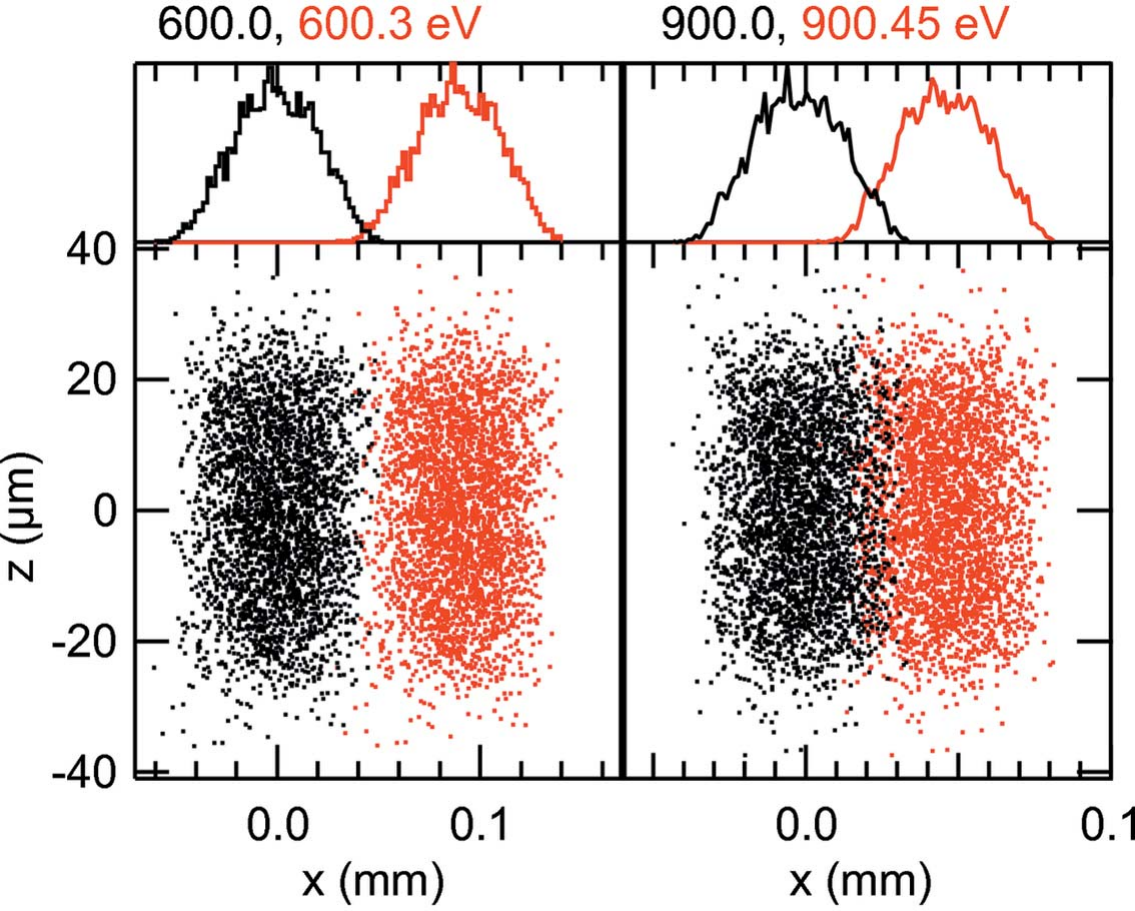
Ray tracings at the exit slit plane. Right-hand panel: 900.00 and 900.45 eV; left-hand panel: 600.0 and 600.3 eV. Histograms at the top show the distributions for each photon energy.

The right-hand panel in Fig. 9 ▶ shows the ray tracings at the exit slit plane obtained with the MEG when the monochromator is tuned to 900 eV and the entrance slit is set to 20 µm horizontal and 36 µm vertical. As seen in the figure, the two energies 900 and 900.45 eV are well resolved showing that the resolution of the monochromator (due to the contributions to the entrance slit, slope errors on the mirror and the grating) at this energy is better than 0.45 eV. This is consistent with the value shown in Fig. 8 ▶, 0.47 eV at 900 eV, which also includes the contribution of the exit slit. We recall that the defocus, coma and spherical aberrations were zeroed at this energy with the VLS parameters. The left-hand panel in Fig. 9 ▶ shows the ray tracings at the exit slit plane at 600 and 600.3 eV under the same conditions as before but when the MEG is tuned to 600 eV. Here the resolution is much better than 0.3 eV. Actually, the calculations shown in Fig. 8 ▶, which include the exit slit contribution, give a total energy resolution of 0.24 eV at 600 eV. Additional ray tracings with this grating at 1200 eV show that at this energy the energy resolution is close to 0.6 eV. The analytical calculations at 1200 eV without the contribution of the exit gives 0.61 eV and with the exit slit the result is 0.78 eV. Similar agreement between the analytical calculations and the ray tracings were also obtained for the other two gratings at several energies in their respective ranges. Based on these results, one can conclude that the line density optimization at one energy for each one of the gratings keeps the aberrations negligible relative to the other terms over the full energy range of each grating.

**Figure 10:**
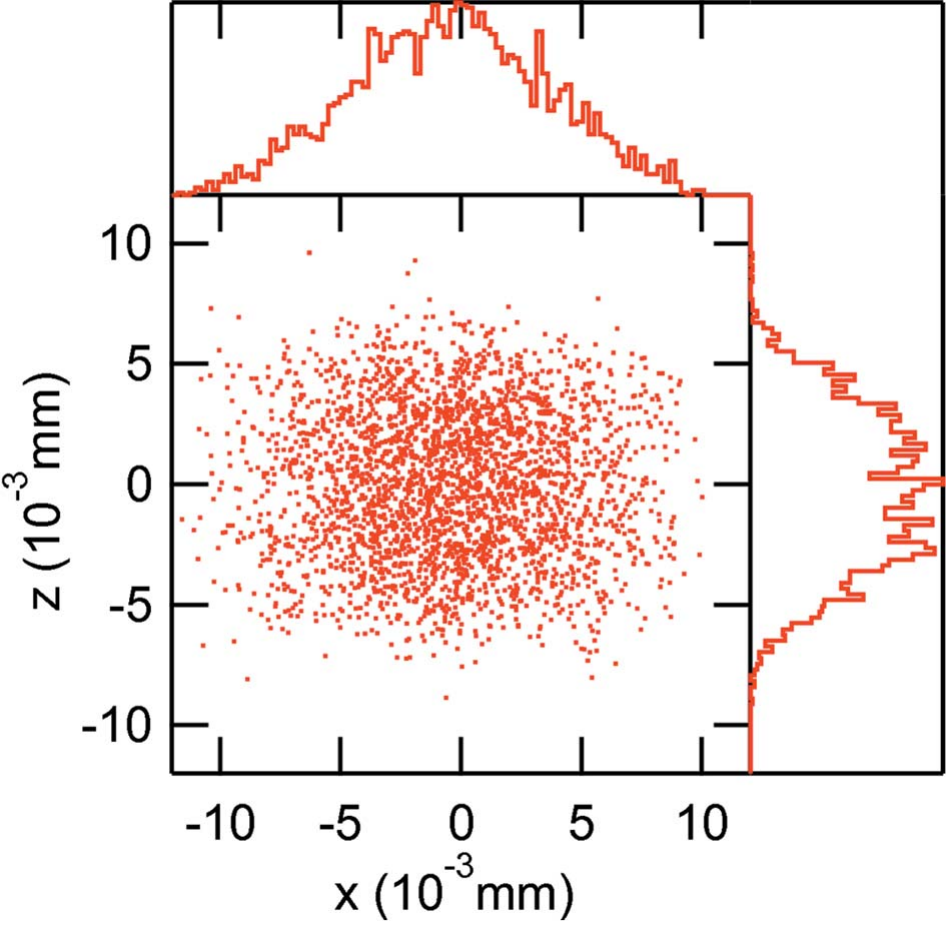
Ray tracings at the sample position at 900 eV with a 20 µm entrance slit (resolution) and a 50 µm exit slit. The pulse duration is 2.3 ps.

The expected spot size at the exit slit when the time slit is set to 2.3 ps (FWHM) and the resolution slits are set to 20 µm (entrance) and 50 µm (exit) can be seen in Fig. 10 ▶. The time broadening due to the illuminated number of lines is ∼0.3 ps, leaving the time resolution practically unchanged. Evidently, the spot size will increase along the vertical direction when the time slit is set to a larger pulse length while keeping the time correlation between vertical position and time. For exit slit sizes larger than approximately 50 µm the horizontal spot size is approximately proportional to the exit slit size and it is sub-linear at smaller openings due to the meridional slope errors on the focusing mirror downstream of the exit slit.

**Figure 11:**
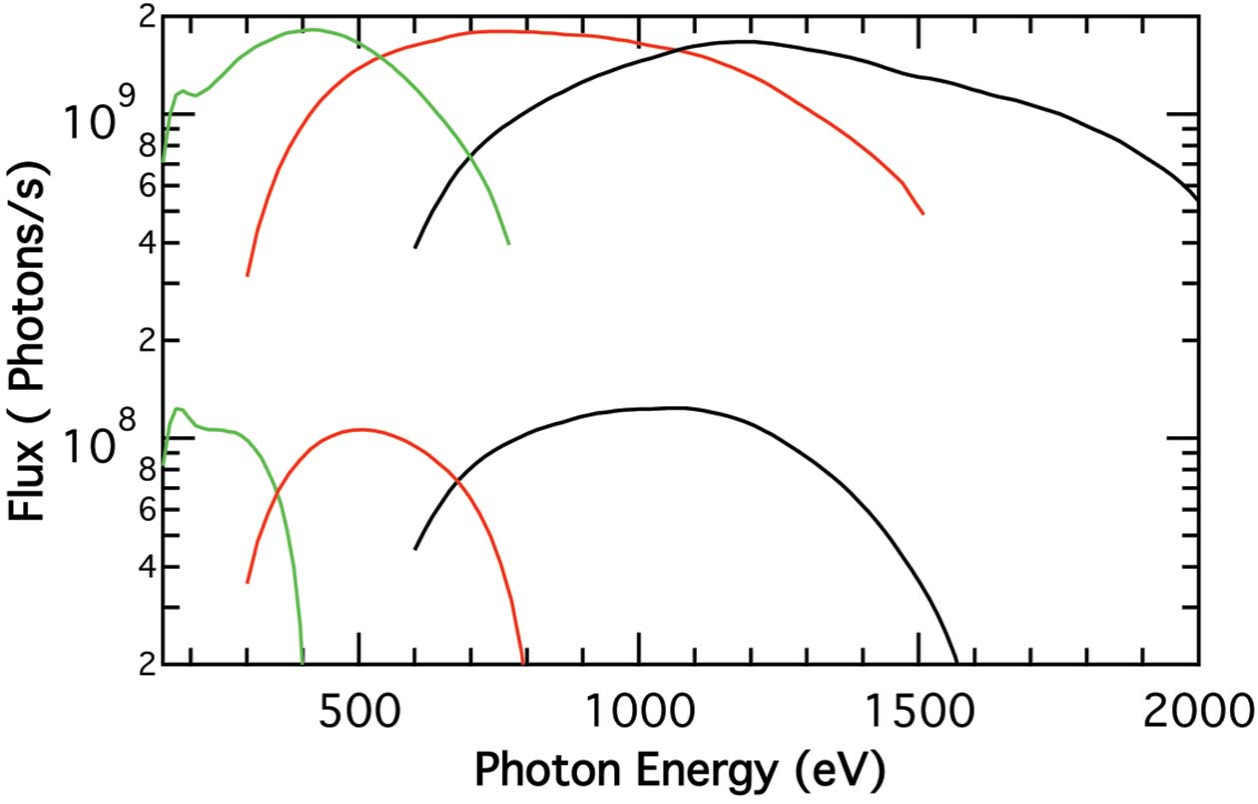
Calculated flux at the sample position with the three gratings for *E*/Δ*E* equal to 2000 and 5000 (lower flux traces). The pulse duration is less than 2.4 ps.

The flux at the sample position as a function of the photon energy was calculated when the time slit was set to a pulse length of 2.3 ps FWHM, taking into account the flux emitted by the bending magnet (Fig. 1 ▶), the reflectivity of the three mirrors assuming they are Au coated, the efficiency of the gratings assuming they are gold coated and that the LEG, MEG and HEG are blazed at 0.51°, 0.56° and 0.68°, respectively. These calculations were performed by adjusting the entrance and exit slits to provide the same resolution and such that *E*/Δ*E* = 2000 and *E*/Δ*E* = 5000. The calculated flux curves (Fig. 11 ▶) show that more than 10^9^ photons s^−1^, or 150 photons pulse^−1^ in the 24-bunch mode of the APS operation, will reach the sample with *E*/Δ*E* = 2000 for photon energies between 150 and 1750 eV. More than one order of magnitude lower flux, ∼6 × 10^7^ photons s^−1^, will reach the sample for energies between 150 and 1360 eV when *E*/Δ*E* = 5000. The pulse duration, including the contribution due to the number of illuminated lines, is less than 2.4 ps for the flux values shown in Fig. 11 ▶. The flux values will be ∼0.4 times smaller when delivering more than 86% of either right or left circular polarized radiation.

## 5. Conclusion   

The SPSXS beamline has been designed to preserve the pulse duration and provide the maximum possible flux to the sample with moderate resolving power. The use of horizontal deflections with sagittal focusing in the vertical direction (time axis) allows the time resolution to be kept down to 1.5 ps (FWHM). Moderate resolving power and high flux is achieved with a horizontally dispersing VLS grating illuminated in converging light and two additional optical elements. The optical design allows the pulse duration to be varied from 1.5 to 100 ps without affecting the energy resolution and the resolution to be changed with negligible effect on the pulse duration.

## Figures and Tables

**Table 1:** Position of the optical elements relative to the center of the bending magnet

Element	Position (m)	Deflection/orientation
Polarization aperture	23.5	
Ellipsoidal mirror	24.8	3° outboard
Time slit	34.8	Vertical
Energy resolution slit	34.8	Horizontal
Experimental station 1	34.9	
Toroidal mirror	38.3	3° inboard
Gratings	38.9	4° inboard
Slit	42.4	Horizontal
Ellipsoidal mirror	46.4	3° outboard
Experimental station 2	47.4	

**Table 2:** Grating parameters

	LEG	MEG	HEG
Range (eV)	150–1200	300–2000	600–2000
*k*_0_ (mm^−1^)	250	500	1000
*a*_1_ (mm^−2^)	0.143	0.286	0.571
*a*_2_ (×10^−4^ mm^−3^)	0.29	0.56	1.2
*a*_3_ (×10^−7^ mm^−4^)	−1.2	−2.4	−4.7
